# Patterns of distant metastases in 215 Merkel cell carcinoma patients: Implications for prognosis and surveillance

**DOI:** 10.1002/cam4.2781

**Published:** 2019-12-27

**Authors:** Christopher W. Lewis, Jamiluddin Qazi, Daniel S. Hippe, Kristina Lachance, Hannah Thomas, Maclean M. Cook, Ilsa Juhlin, Neha Singh, Zoe Thuesmunn, Seesha R. Takagishi, Aubriana McEvoy, Coley Doolittle‐Amieva, Shailender Bhatia, Kelly G. Paulson, Ryan B. O'Malley, Carolyn L. Wang, Paul Nghiem

**Affiliations:** ^1^ Division of Dermatology Department of Medicine University of Washington Seattle WA USA; ^2^ Division of Medical Oncology Department of Medicine University of Washington Seattle WA USA; ^3^ Department of Radiology University of Washington Seattle WA USA; ^4^ Seattle Cancer Care Alliance Seattle WA USA

**Keywords:** carcinoma, Merkel cell, dermatology, medical oncology, neoplasm metastasis, neoplasm staging, prognosis, radiology

## Abstract

Approximately one‐third of Merkel cell carcinoma (MCC) patients eventually develop distant metastatic disease. Little is known about whether the location of the primary lesion is predictive of initial distant metastatic site, or if survival likelihood differs depending on the metastatic site. Such data could inform imaging/surveillance practices and improve prognostic accuracy. Multivariate and competing‐risk analyses were performed on a cohort of 215 MCC patients with distant metastases, 31% of whom had two or more initial sites of distant metastasis. At time of initial distant metastasis in the 215 patients, metastatic sites (n = 305) included non‐regional lymph nodes (present in 41% of patients), skin/body wall (25%), liver (23%), bone (21%), pancreas (8%), lung (7%), and brain (5%). Among the 194 patients who presented with MCC limited to local or regional sites (stage I‐III) but who ultimately developed distant metastases, distant progression occurred in 49% by 1 year and in 80% by 2 years following initial diagnosis. Primary MCC locations differed in how likely they were to metastasize to specific organs/sites (*P* < .001). For example, liver metastases were far more likely from a head/neck primary (43% of 58 patients) versus a lower limb primary (5% of 39 patients; *P *< .0001). Skin‐only distant metastasis was associated with lower MCC‐specific mortality as compared to metastases in multiple organs/sites (HR 2.7; *P* = .003), in the liver (HR 2.1; *P* = .05), or in distant lymph nodes (HR 2.0; *P* = .045). These data reflect outcomes before PD1‐pathway inhibitor availability, which may positively impact survival. In conclusion, primary MCC location is associated with a pattern of distant spread, which may assist in optimizing surveillance. Because it is linked to survival, the site of initial distant metastasis should be considered when assessing prognosis.

## Graphical abstract

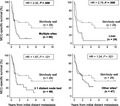

## 1 INTRODUCTION

Merkel cell carcinoma (MCC) is an aggressive skin cancer with a 5‐year disease‐associated mortality of 40%.^1^ Risk factors for MCC include age >50, ultraviolet light exposure, Caucasian race, immune suppression, and the Merkel cell polyomavirus.^2^, ^3^ About 2500 cases of MCC are reported in the United States each year and this incidence is increasing.^4^, ^5^, ^6^

Merkel cell carcinoma has a high propensity to recur. The characteristics of local and regional MCC recurrences are well described in the literature.^7^, ^8^, ^9^ However, data regarding the timing and pattern of distant MCC metastases are scarce limited to case reports and small series.^10^ Therefore, existing surveillance guidelines for metastatic MCC are not evidence based, which leads to vague and inconsistent management recommendations across institutions.^11^, ^12^ Evidence‐based standardization of surveillance practices could facilitate efficient use of resources and earlier detection of metastases.

Historically, early detection of metastasis was not prioritized in MCC management. Until the development of immunotherapies (ie, anti‐PD1/PDL1), the standard of care for metastatic disease was chemotherapy. Responses to chemotherapy were rarely durable, even if the metastatic disease burden was small, so early detection of MCC spread did not improve survival.^13^ With the advent of immunotherapies and more durable treatment responses,^14^, ^15^ early identification of metastases could improve response and survival rates. A comprehensive analysis of metastatic patterns would inform such surveillance practices.

In the current study, we performed a retrospective analysis of 215 patients who developed distant metastatic MCC. We investigated the prognostic and clinical significance of the initial MCC metastatic sites.

## 2 METHODS

### 2.1 Patients

**Figure 1:**
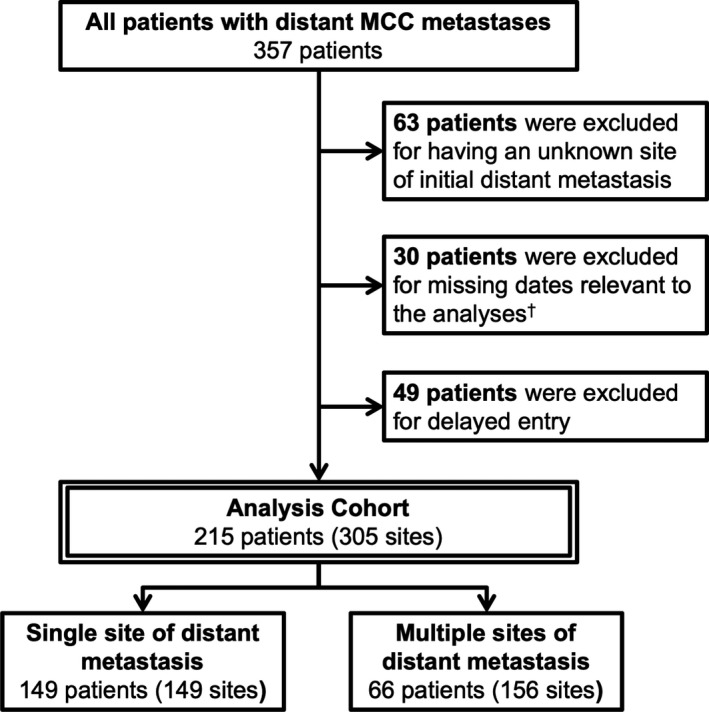
Flowchart of Merkel cell carcinoma (MCC) patient selection for Analysis Cohort. Patients in the Analysis Cohort either presented with stage IV MCC or developed distant metastases during follow‐up and had sufficient data to identify the location and timing of their distant metastases. The 49 patients excluded for delayed entry enrolled either greater than 180 days after their initial distant metastasis or were enrolled after death (13 patients were enrolled by family members or legal representative after their death). ^†^Dates required for analysis were date of initial distant metastasis, date of death or last follow‐up, and date of initial consent

Persons with pathologically confirmed MCC were enrolled in an IRB‐approved repository of data and specimens. Each patient provided written informed consent. These data included 1168 MCC patients enrolled between November 2000 and March 2016 and monitored longitudinally during this same period. Of these patients, 357 were diagnosed with distant MCC metastases or developed distant metastases after initial therapy. Patients were excluded if initial distant metastatic site (n = 63) or detection date were unavailable (n = 30) or if they were enrolled after death (n = 13). Patients were also excluded if they enrolled more than 180 days after initial metastatic diagnosis (n = 36, Figure 1), to avoid inadvertent selection bias. The remaining 215 patients had sufficient information to determine the site of initial distant metastasis and survival outcomes. Merkel cell polyomavirus (MCPyV) status was determined either using immunohistochemistry or oncoprotein antibody status.^16^ The data that support the findings of this study are available from the corresponding author upon reasonable request.

### 2.2 Classifying initial distant metastases

The American Joint Committee on Cancer (AJCC) 8th edition staging criteria^17^ were followed to define distant metastases as any clinically, pathologically, or radiologically confirmed MCC found beyond the skin‐draining lymph nodes of the primary lesion site. In patients without a known primary lesion, metastases were considered “distant” if they involved visceral sites, or node beds not directly draining skin (eg, mediastinal or retroperitoneal), or multiple skin‐draining nodes beds that were not contiguous (eg, parotid and popliteal). Radiology data were mostly taken from the scan reports. Uncertain cases were reviewed by University of Washington radiologists to confirm metastatic sites. Pathology data were gathered in cases with biopsy‐confirmed distant metastases.

### 2.3 Grouping sites of initial distant metastases

Sites of distant metastasis included: distant lymph nodes, distant skin/body wall, liver, bone, pancreas, brain, adrenal gland, bowel, peritoneum/retroperitoneum, gonad, oral, heart, kidney, spleen, and urinary bladder. In addition to cutaneous lesions, the “skin/body wall” site included superficial lesions found in the breast, muscle, and soft tissues. The “bowel” category included intramural or intraluminal metastases to the stomach, large intestine, and small intestine, whereas “peritoneum/retroperitoneum” included the bowel surface or serosa. Patients were categorized by the presence or absence of a distant metastasis in each organ site group. Sites of initial distant metastasis were also grouped by AJCC 8th edition substages for comparison, specifically as skin/lymph node only (M1a), lung (M1b), and any other site (M1c).

### 2.4 Statistical analyses

For analysis relating MCC primary site to sites of metastasis, patients were first grouped by their primary site. For each primary site group, the frequency of metastases among a series of sites was calculated. The pattern of metastasis among patients with that primary site was compared to the metastatic pattern of all other primary sites combined using a permutation test. For each significant comparison, a post‐hoc Fisher's exact test was used to identify which individual distant metastatic sites differed between the groups.

The survival analysis primary endpoint was MCC‐specific survival. All other causes of death were considered a competing risk. Survival time was the interval from first detection of initial distant metastasis to last follow‐up date or death. Patients who died of an unknown cause were censored at the time of death. Univariate and multivariate Fine and Gray competing‐risks regression models^18^ were used to evaluate associations between risk of MCC‐specific death and distant metastatic site, primary tumor site, stage at diagnosis, sex, age at first distant metastasis, and immunosuppression status. MCPyV status was not included in the multivariate analysis because it was available in 109 of 215 patients and it did not independently add predictive value to metastatic pattern analyses or survival outcomes. Left truncation was used to account for patients who were enrolled after the day of their first metastatic diagnosis.

For the survival analyses, sites of initial distant metastasis were grouped into mutually exclusive categories: skin/body wall, distant lymph nodes, liver, all other visceral sites, and multiple sites. The multiple sites category was used when a patient had distant metastases at multiple sites within otherwise different categories (eg, skin/body wall and distant lymph nodes). All statistical analyses were performed using Stata (version 14) and R (version 3.1.1). Throughout, two‐sided significance threshold of 5% was used without adjustment for the number of comparisons.

## 3 RESULTS

### 3.1 Patients

Two hundred and fifteen patients with metastatic MCC met inclusion criteria. These patients had 305 sites of metastatic disease when initially diagnosed with distant metastases. Patients enrolled at various stages of management, including 78 (36%) patients who developed distant metastases prior to enrollment. Most patients had initial metastatic disease limited to a single site (n = 149, 69%). Patients with multiple sites (n = 66) most often had metastatic disease in two sites (median 2; range 2‐5; Table 1). Radiology results of initial distant metastases were reviewed for all 215 patients. 109 (51%) patients had a pathologically confirmed distant metastasis in addition to imaging.

**Table 1:** Clinical characteristics of Merkel cell carcinoma Analysis Cohort (n = 215 patients). All clinical characteristics were captured at initial diagnosis except age, which was recorded at initial distant metastasis

	n	%
Sex		
Male	176	82
Female	39	18
Age at first distant met		
≤65	68	32
>65	147	68
Primary lesion site		
No identified primary	42	20
Head and neck	58	27
Trunk	19	9
Buttock^a^	11	5
Upper limb	46	21
Lower limb	39	18
Stage at diagnosis		
Local	78	36
Nodal	116	54
Distant	21	10
Immune suppression		
Present	43	20
Absent	172	80
Number of metastatic sites		
One	149	69
Two	46	21
Three	17	8
Four	2	<1
Five	1	<1
MCPyV status^b^		
Positive	94	44
Negative	15	7
Unknown	106	49

a The buttock category includes one patient with a genital primary lesion.

b One hundred and nine of the 215 patients had known Merkel cell polyomavirus (MCPyV) tumor status as determined by immunohistochemistry or oncoprotein antibody status.

**Figure 2:**
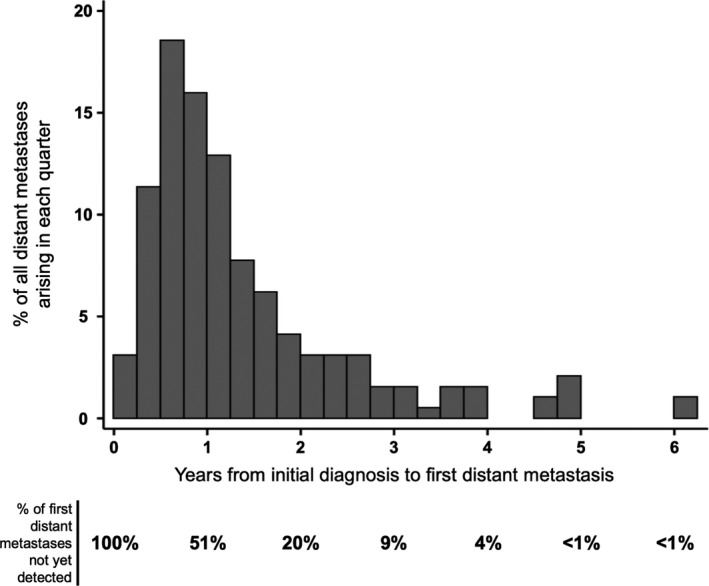
Timing of initial distant metastases after diagnosis (n = 194 patients). Patients in the Analysis Cohort (n = 215) who presented with distant metastases at initial diagnosis (n = 21) were excluded from this analysis. Patients were grouped by the quarter year in which they developed their first distant metastasis. The height of each bar is the percentage of patients who developed distant metastases during that quarter. On the bottom of the figure is listed the percentage of patients who have yet to develop their initial distant metastasis at the beginning of each year

One hundred and ninety‐four of the 215 patients developed distant metastases after initial staging (stage I‐III). Among this subset of patients, the median time between primary MCC diagnosis and first distant metastasis was 385 days (1.05 years). The percentage of patients who developed distant metastases by the end of 1‐, 2‐, and 5‐ years were 49%, 80%, and 99%, respectively (Figure 2). Neither primary MCC site nor location of distant metastasis was predisposed to early or late development of distant metastatic disease.

### 3.2 Association between clinical characteristics and initial metastasis sites

**Figure 3:**
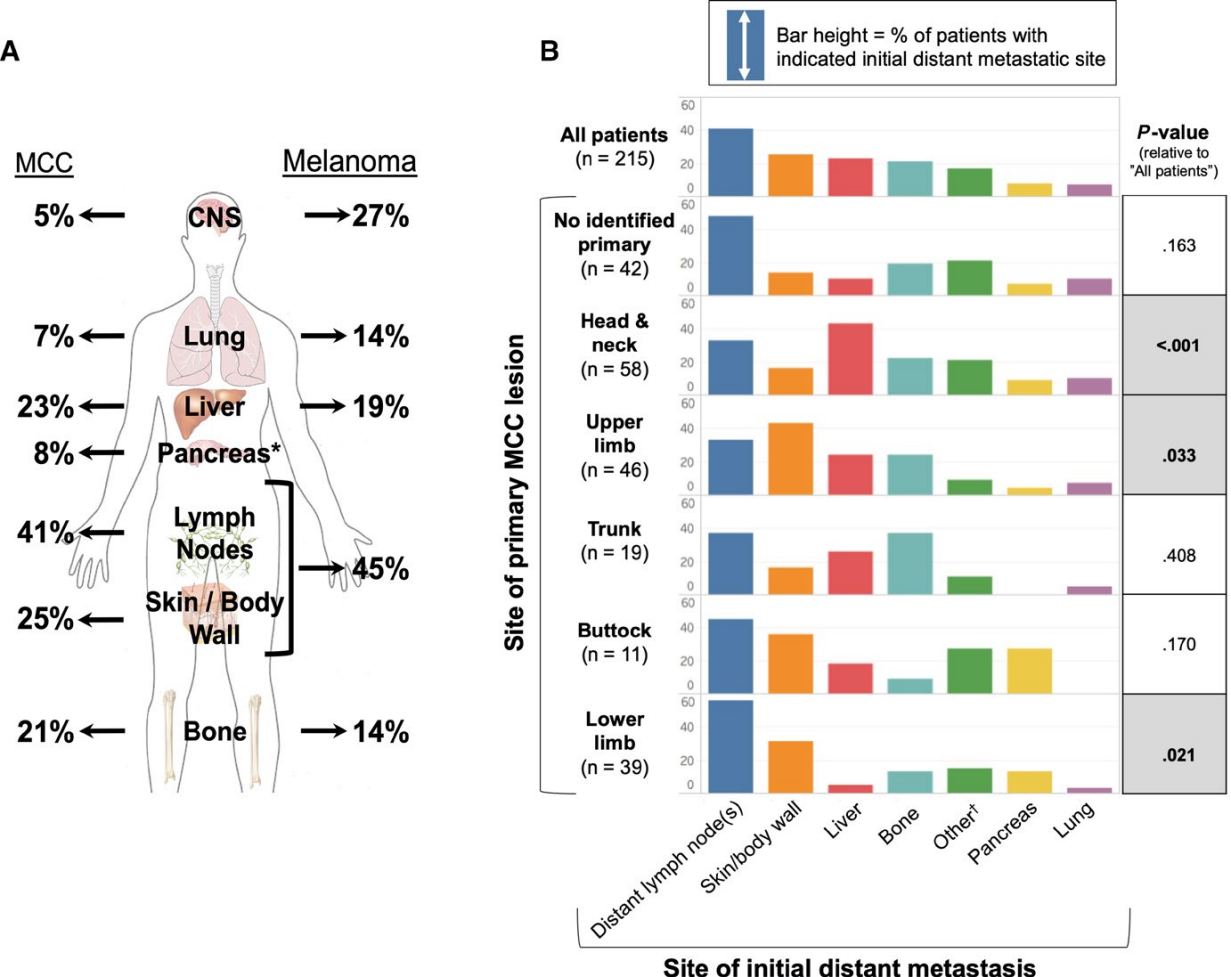
A, Comparison of sites of initial distant metastasis between Merkel cell carcinoma (MCC) and melanoma. Percentages shown indicate the fraction of patients (with MCC or melanoma as indicated) whose first distant metastatic disease presented at each listed anatomic site. Given that some patients' initial metastatic disease was apparent at multiple sites, percentages totaled to greater than 100%. Melanoma data were obtained from multiple publications^19^, ^29^ with Samlowski et al reporting central nervous system (CNS) metastases and Tas et al reporting all others. Data for MCC metastases are from the 215 patients included in this study. Metastatic frequency by anatomic site was not compared for statistical significance as the melanoma data were aggregated from multiple cohorts. ^*^There were not ample data describing the rate of initial metastatic involvement of the pancreas in patients with metastatic melanoma. B, Pattern of initial site of distant metastasis by site of primary lesion (215 patients; 305 sites of metastasis). Patients were grouped by their primary Merkel cell carcinoma site. Each bar represents the percentage of patients with a given primary site who developed an initial distant metastasis in the indicated distant site. 66 patients had multiple sites of initial distant metastasis (157 sites) and could contribute to multiple bars in one row (the median number of sites among patients with multiple sites was 2). As a result, the sum of each row is greater than 100%. A patient could not contribute to multiple bars in any column, as each patient had only one primary site. The metastatic site frequencies for each primary lesion site group (each row) were compared with the metastatic site frequency of all other primary site groups with the corresponding p‐value representing the significance of this comparison. ^†^The “other” category includes brain, adrenal gland, bladder, bowel, gonad, heart, kidney, peritoneum/retroperitoneum, oral, and spleen

Initial distant metastases were frequently found in distant lymph nodes (88 of 215 patients, 41%), skin/body wall (54, 25%), liver (49, 23%), bone (45, 21%), pancreas (18, 8%), and lung (15, 7%) (Figure 3A). Less frequent initial distant metastatic sites included brain (11, 5%), peritoneum/retroperitoneum (7, 3%), bowel (6, 3%), adrenal glands (5, 2%), gonad (5, 2%), spleen (2, 1%), kidneys (2, 1%), heart (1, <1%), and oral cavity (1, <1%). Groupings were not mutually exclusive as 66 patients had 2 or more concurrent sites.

Location of the initial distant metastasis was significantly associated with location of the primary lesion (*P* < .001, Figure 3B). Notably, patients with a head/neck primary site were more likely to develop a liver metastasis (43.1% vs 15.3%, *P* < .001) and less likely to develop a distant skin/body wall metastasis (15.5% vs 28.7%, *P* = .053) compared to patients with any other primary site. In contrast, liver metastases were less common in patients with lower limb primaries than other primary sites (5.1% vs 26.7%, *P* = .003) and distant skin/body wall metastases were more common in patients with an upper limb primary than any other primary site (43.5% vs 20.1%, *P* = .002). Patients with lower limb primaries were also more likely to have a distant nodal metastasis (56.4% vs 37.5%, *P* = .033) than those with any other primary site.

### 3.3 Survival estimates

A total of 163 of 215 (75%) patients died during follow‐up (547 total patient‐years; median 1.9 years): 145 died of MCC, 6 died of non‐MCC causes, and 12 lacked adequate clinical information to determine cause. After diagnosis of initial distant metastases, the 1‐, 3‐, and 5‐year MCC‐specific survival were 45%, 22%, and 18%, respectively, with a median MCC‐specific survival time of 359 days.

**Figure 4:**
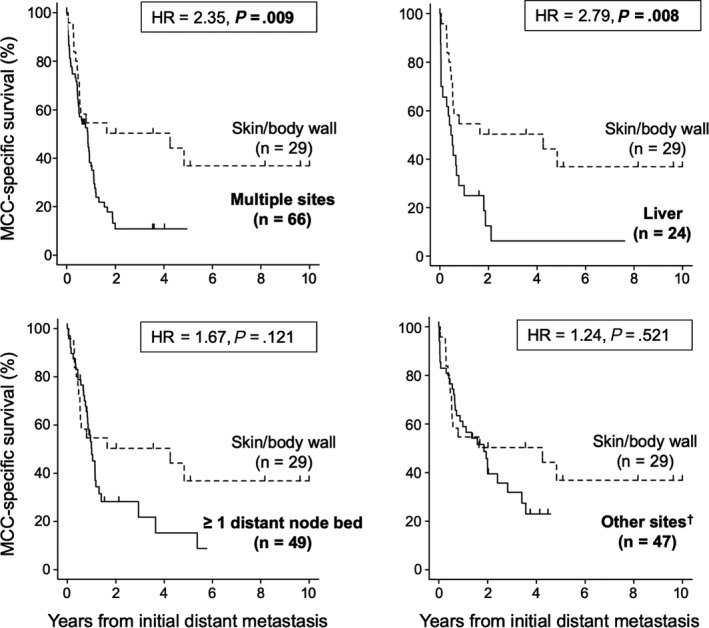
Merkel cell carcinoma (MCC) specific survival comparison by site of first distant metastasis (n = 215 patients). Five aggregate categories for initial metastatic site were created for statistical analysis. Twenty‐nine patients developed initial distant metastases limited to the skin/body wall (dashed line). The MCC‐specific survival of all other sites of initial distant metastasis (solid lines) was compared to the survival of patients with skin‐only distant metastases. Tick marks indicate patient censoring. Hazard ratios and corresponding p‐values were calculated using univariate competing‐risks regression models. Complete univariate and multivariate competing‐risks estimates are described in Table 2. ^†^The “other” category includes brain, adrenal gland, bladder, bowel, gonad, heart, kidney, peritoneum/retroperitoneum, oral, and spleen

Patients with multiple sites of distant metastases (n = 66) were separated from patients with a single site (n = 149). The 149 patients with a single site were grouped by most common site: distant lymph nodes (n = 49), distant skin/body wall (n = 29), and liver (n = 24). All other sites were combined (n = 47) for survival analysis. Patients with multiple metastatic sites had lower 1‐, 3‐, and 5‐year survival rates (36%, 10%, no data) compared with patients with a single site (49%, 27%, 18%) (HR = 1.54, 95% CI 1.12‐2.21, *P* = .010). Furthermore, single metastatic site subgroupings had significantly different MCC‐specific survival outcomes (*P* = .027): skin/body wall (1‐, 3‐, and 5‐year survival: 47%, 47%, 32%), lymph nodes (47%, 13%, 7%), liver (25%, 6%, 6%), and other visceral sites (59%, 30%, no data; Figure 4; Table 2 **)**.

**Table 2:** The relationship between Merkel cell carcinoma (MCC) specific survival and clinical characteristics (215 patients, 145 MCC‐specific deaths). Survival analysis was completed using a competing‐risks regression (non‐MCC causes of death were considered competing events). Stage at diagnosis (*P* = .53 univariate; *P* = .56 multivariate) and age at first metastasis (*P* = .51; *P* = .31) were not significantly associated with MCC‐specific survival (data not shown)

	Univariate			Multivariate^a^					
				Grouped by most frequent initial metastatic sites			Grouped by M1 AJCC 8th edition substaging		
	HR	95% CI	Global *P*‐value	HR	95% CI	Global *P*‐value	HR	95% CI	Global *P*‐value
Site of initial metastasis			.006			.009			
Skin/ body wall (ref)	—	—		—	—				
Lymph node	1.67	0.87‐3.20		1.97	1.01‐3.84				
Liver	2.79	1.31‐5.96		2.13	1.00‐4.55				
All other visceral sites	1.24	0.64‐2.42		1.26	0.64‐2.49				
Multiple sites	2.35	1.24‐4.45		2.73	1.41‐5.31				
Met site by AJCC substaging			.075						.170
M1a: skin/ lymph node (ref)	—	—					—	—	
M1b: lung	0.89	0.42‐1.86					0.77	0.37‐1.60	
M1c: all other sites	1.44	1.02‐2.04					1.29	0.90‐1.87	
Immune suppression			<.001			<.001			<.001
Absent (ref)	—	—		—	—		—	—	
Present	1.97	1.35‐2.88		2.15	1.41‐3.27		2.10	1.42‐3.11	
Primary lesion site			.023			.215			.237
No identified primary (ref)	—	—		—	—		—	—	
Head and neck	1.53	0.98‐2.37		1.09	0.62‐1.93		1.38	0.80‐2.39	
Trunk	2.12	1.26‐3.58		1.18	0.54‐2.57		1.43	0.72‐2.85	
Buttock & genitalia	0.97	0.46‐2.05		0.59	0.26‐1.36		0.75	0.34‐1.66	
Upper limb	1.43	0.86‐2.39		1.11	0.61‐2.02		1.29	0.73‐2.26	
Lower limb	0.90	0.55‐1.48		0.63	0.35‐1.15		0.80	0.44‐1.43	
Patient sex			.080			.031			.046
Male (ref)	—	—		—	—		—	—	
Female	0.67	0.42‐1.05		0.55	0.32‐0.95		0.59	0.35‐0.99	

a The multivariate analyses included the following variables: site(s) of first distant metastasis, primary lesion site, sex, immune suppression status, age at first metastasis, and stage at diagnosis.

Site of initial distant metastasis remained significantly associated with prognosis after adjustment for primary lesion site, stage at diagnosis, immune suppression, sex, and age (*P* = .009, Table 2). Specifically, multiple metastatic sites (HR = 2.73, 95% CI 1.41‐5.31, *P* = .003), liver metastases (HR = 2.13, 95% CI 1.00‐4.55, *P* = .050), and nodal metastases (HR = 1.97, 95% CI 1.02‐3.84, *P* = .045) were associated with worse prognosis than distant skin/body wall metastases after multivariate adjustment. Immune suppression (*P* < .001) and male sex (*P* = .031) were also associated with worse prognosis after multivariate adjustments. Primary lesion site was significantly associated with prognosis in univariate analysis (*P* = .022), but not after adjusting for other factors (*P* = .215, Table 2). Notably, the prognosis of patients diagnosed with metastatic disease at initial presentation (n = 21) was not significantly different than that of patients diagnosed with distant metastasis during follow‐up (n = 194; HR 0.76, CI 0.44‐1.29, *P* = .307), though the stage IV group was small.

An alternative grouping of initial distant metastatic sites was examined based on AJCC 8th edition MCC substages. Of the 215 patients, there were 86 (40%) with distant metastases to skin, subcutaneous tissue, or lymph nodes (M1a), 7 (3%) with lung metastases (M1b), and 122 (57%) with metastases at other sites (M1c). When compared with the M1a group, patients in the M1c group were more likely to die of MCC in a univariate analysis (HR 1.44, 95% CI 1.02‐2.04, *P* = .039). However, the survival difference was not significant in multivariate analysis (HR 1.29, 95% CI 0.90‐1.87 *P* = .170; Table 2).

## 4 DISCUSSION

This study explored relationships between MCC primary and distant metastatic sites as well as associated survival outcomes by metastatic site. Initial distant MCC metastases included nearly every organ and anatomic site in the body, and most commonly distant lymph nodes, skin/body wall, liver, bones, lung, and pancreas. Distant metastases typically presented relatively soon after initial diagnosis, with 80% of first distant metastases arising within 2 years of diagnosis (Figure 2). Patterns of initial metastatic sites in MCC were distinct from melanoma,^19^ particularly with respect to involvement of the lungs, brain, and pancreas (Figure 3A). Primary MCC site indicated relative risk of spread to specific distant metastatic sites. Subsequent disease‐specific survival was significantly associated with the site of distant metastasis—with initial metastases to multiple sites, liver, and lymph nodes being associated with poorer prognosis when compared with skin‐only metastatic disease.

A recent study reviewing the site and timing of distant metastases among 30 MCC patients found metastatic patterns similar to this study.^10^ Distribution of metastatic sites was similar between studies, however, Kouzmina et al did not associate metastatic site with initial primary site or discuss survival differences by metastatic site.

The prevalence of distant metastatic disease has been described in many prior reports.^1^, ^17^ To the best of our knowledge, existing literature has not significantly addressed (a) a MCC‐specific metastatic pattern dependent on primary site and (b) the relationship between survival and MCC spread to specific distant sites.

Individual primary sites were associated with specific patterns of initial distant metastatic site. Patients with a head/neck primary lesion were far more likely to have initial distant metastases in the liver than any other primary site. Early screening for liver metastases among head/neck primary patients may improve survival because liver metastases were associated with an increased mortality in the chemotherapy era. However, immunotherapy will likely improve outcomes for any distant metastatic site as it can lead to durable responses.^20^ We also examined whether primary sites had significantly different chances of developing any distant metastasis and found no site with significantly higher or lower risk (data not shown). Thus, the primary site was associated with the location of distant metastatic spread, but not associated with the overall likelihood of developing distant disease.

The AJCC 7th and 8th edition MCC distant metastasis substaging systems (stage IV) were extrapolated from the 7th edition melanoma criteria. Specifically, skin, subcutaneous, tissue, and lymph nodes were categorized as substage M1a, lung (M1b), all other distant sites (M1c).^1^, ^17^, ^21^, ^22^ Indeed, these groups stratify prognosis for metastatic melanoma, but were not associated with significant survival differences in our MCC cohort (Table 2). Assuming an independent cohort has similar findings, it may be appropriate to revise MCC stage IV subgroups based on MCC‐specific data rather than melanoma‐based data.

Due to significant survival differences, AJCC 8th edition included a new subgrouping for melanoma metastases to the central nervous system (M1d).^17^, ^21^, ^22^ Our study does not support similar changes to MCC substaging because brain metastases are much less common among MCC patients (5% MCC and 27% melanoma, Figure 3B) and the small sample of MCC brain metastases precluded statistical evaluation of survival data for this category.

Until recently, chemotherapy was the only systemic treatment option for metastatic MCC. While tumor volume is initially reduced by chemotherapy in over half of patients, responses are not durable and progression typically occurs within 3 months of starting chemotherapy.^13^, ^23^ Conversely, multiple trials of immune checkpoint inhibitors in skin cancers have proven to be effective^15^, ^20^, ^24^, ^25^ and there is evidence that they are most effective when disease burden is small.^26^ This highlights the importance of using MCC‐specific metastatic patterns to detect disease spread early and provide rescue therapies before patients developed more extensive disease. In our cohort, the median time to first recurrence was less than a year, suggesting more frequent surveillance during this period could detect metastases earlier and improve the rate and/or duration of responses to immunotherapy.

Prior to development of distant metastatic disease, initial therapy of the patients in this study cohort typically followed National Comprehensive Cancer Network guidelines.^11^ One exception was that patients treated at our facility who had risk factors associated with local and regional MCC recurrence were more likely to receive adjuvant radiation than is typical, according to data from the National Cancer Database.^27^ Overall, adjuvant radiation is known to decrease local and regional recurrence risk but has little or no effect on the risk of developing distant metastatic disease.^28^ Thus we suspect that distant metastatic risk in this cohort will be representative of the behavior of this disease more broadly in the United States.

This study had relevant limitations. This cohort did not include patients who received checkpoint inhibitor treatment; therefore, we do not know how these therapies would affect metastatic patterns and survival statistics. Finally, imaging modality and surveillance frequency were inconsistent, though this represented real‐world variation.

In conclusion, MCC has a unique pattern of initial distant metastases, which appears to differ from that of melanoma. Initial distant metastatic site of MCC is a relevant predictor of survival. Using MCC‐specific metastatic patterns should contribute to subsequent improvements in surveillance guidelines and prognostic accuracy.

## CONFLICT OF INTEREST

Chris Lewis received a grant from the American Cancer Society to perform this research. Kelly Paulson reports receiving research grants from SITC‐Merck, Bluebird Bioscience, and EMD‐Serono. Shailender Bhatia reports receiving grants from BMS, Merck, Novartis, EMD‐Serono, Oncosec, Immune Design, and NantKwest, as well as honoraria (for advisory board participation) from BMS, EMD‐Serono, and Sanofi‐Genzyme. Paul Nghiem reports receiving grant support from EMD Serono and Bristol Myers Squibb as well as honoraria from Merck and EMD‐Serono. Dan Hippe reported being a collaborator on industry‐funded work with GE Healthcare, Philips Healthcare, Toshiba America Medical Systems, and Siemens Medical Solutions USA. Ryan O'Malley reports receiving grant support from GE Healthcare. None of these disclosures were directly related to this publication.

## AUTHOR CONTRIBUTIONS

Christopher W. Lewis was involved in conceptualization, data curation, formal analysis, funding acquisition, investigation, methodology, project administration, validation, visualization, writing—original draft, and writing—review and editing. Jamiluddin Qazi was involved in conceptualization, data curation, formal analysis, funding acquisition, investigation, methodology, validation, visualization, writing—original draft, and writing—review and editing. Daniel S. Hippe was involved in formal analysis, investigation, validation, visualization, writing—original draft, and writing—review and editing. Kristina Lachance was involved in data curation, funding acquisition, investigation, project administration, and writing—review and editing. Hannah Thomas and Zoe Thuesmunn were involved in data curation, investigation, and writing—review and editing. Maclean M. Cook and Neha Singh were involved in conceptualization, data curation, formal analysis, investigation, methodology, and writing—review and editing. Ilsa Juhlin was involved in data curation and writing—review and editing. Seesha R. Takagishi was involved in data curation, formal analysis, investigation, validation, and writing—review and editing. Aubriana McEvoy was involved in data curation, formal analysis, validation, visualization, and writing—review and editing. Shailender Bhatia and Kelly G. Paulson were involved in conceptualization, formal analysis, funding acquisition, investigation, methodology, supervision, validation, and writing—review and editing. Ryan B. O'Malley was involved in conceptualization, data curation, investigation, methodology, supervision, validation, and writing—review and editing. Carolyn L. Wang was involved in conceptualization, data curation, formal analysis, investigation, methodology, supervision, validation, and writing—review and editing. Paul Nghiem was involved in conceptualization, data curation, formal analysis, funding acquisition, investigation, methodology, project administration, resources, supervision, validation, visualization, writing—original draft, and writing—review and editing.

## Data Availability

The data that support the findings of this study are available from the corresponding author upon reasonable request.
